# Enclosure of all index-1 saddle points of general nonlinear functions

**DOI:** 10.1007/s10898-016-0430-8

**Published:** 2016-05-05

**Authors:** Dimitrios Nerantzis, Claire S. Adjiman

**Affiliations:** Department of Chemical Engineering, Imperial College London, London, United Kingdom

**Keywords:** Global optimization, Transition states, Interval matrix, Eigenvalue bounding, NP-Hard

## Abstract

Transition states (index-1 saddle points) play a crucial role in determining the rates of chemical transformations but their reliable identification remains challenging in many applications. Deterministic global optimization methods have previously been employed for the location of transition states (TSs) by initially finding all stationary points and then identifying the TSs among the set of solutions. We propose several regional tests, applicable to general nonlinear, twice continuously differentiable functions, to accelerate the convergence of such approaches by identifying areas that do not contain any TS or that may contain a unique TS. The tests are based on the application of the interval extension of theorems from linear algebra to an interval Hessian matrix. They can be used within the framework of global optimization methods with the potential of reducing the computational time for TS location. We present the theory behind the tests, discuss their algorithmic complexity and show via a few examples that significant gains in computational time can be achieved by using these tests.

## Introduction

We consider the following problem: Given a function $f:B \subseteq \mathbb {R}^n \rightarrow \mathbb {R}$, $f \in C^2$ we want to find all the critical points, $x^* \in B:$ $\nabla f (x^*)=0$, of *f* for which the Hessian matrix $\nabla ^2f(x^*)$ has eigenvalues $\lambda _n < 0 < \lambda _{n-1} \le \cdots \le \lambda _1$. Such points are called transition states (TSs) or index-1 saddle points. TSs play a crucial role in determining rates of chemical transformations [28] and are also of interest in robotics and economics [9].

A number of local methods have been proposed in the literature for the identification of transition states. For example, in the rational function optimization (RFO) method [4] and the Dimer method [13], a local search for a single TS is performed, while in the nudged elastic band method [14], an approximation of the minimum energy path between two minima is constructed and a TS is found as the point with the maximum energy on this path. In [8], an alternative approach is based on the transformation of the initial potential energy surface so that TSs correspond to local minima on the new surface. Stochastic methods such as simulated annealing [6] and genetic algorithms [9] have also been employed for locating TSs. While computationally more expensive, such methods do not require any starting points to locate a TS and may find multiple TSs.

Our focus in this paper is on deterministic global methods, that can guarantee the identification of all TSs within a specified domain. In the existing literature, the use of such methods for TS location includes the work of Westerberg and Floudas [29] using the $\alpha$BB algorithm [2, 3] and the work of Lin and Stadtherr [18] using an interval Newton method [11, 22]. In [18, 29] the authors locate all critical points of a potential energy function and then classify the solutions based on the signs of the eigenvalues of the corresponding Hessian matrices. This approach has been found to be reliable but a drawback in the context of TS location is that computational time is spent locating, to a high accuracy, critical points with index greater than 1 (i.e., with a number of negative eigenvalues greater than 1), and index-0 (i.e. minima). Because of the computational cost associated with deterministic global optimization, it may be beneficial to focus the search on regions that contain TSs only. In this paper, we expand on our early work [24] and propose several tests that allow the elimination of certain regions. We apply this approach to a number of test functions. Through these examples, we explore the trade-off between the cost of the tests and the number of iterations and CPU time required to identify all TSs.

The paper is organized as follows: In Sect. 2, we give some basic notions and definitions related to interval matrices. In Sect. 3, we introduce the general algorithmic framework. The regional tests are presented in Sect. 4. Local search over index-1 areas is discussed in Sect. 5. In Sect. 6 we characterize the tests in terms of their completeness. In Sect. 7, we address the algorithmic complexity of the problems that we aim to solve with the tests. The algorithm is applied to a number of examples in Sect. 8 and conclusions are drawn in Sect. 9.

## Preliminaries

We make extensive use of concepts from interval arithmetic throughout this paper. We introduce the necessary concepts in this section and the reader is referred to [22] for further details.

We denote interval variables with lower case letters inside square brackets, [*x*], and the corresponding lower and upper bounds as $\underline{x}$ and $\overline{x}$ respectively. Interval matrices are denoted with capital letters inside square brackets. An interval matrix is simply a matrix with interval entries instead of scalar entries. For example, a symmetric interval matrix is $$[M] = \begin{bmatrix} [-3,-2]&[-0.5,0.5] \\ [-0.5,0.5]&[-4,-3] \end{bmatrix}$$. The interval matrix [*M*] can be interpreted as the infinite set of symmetric scalar matrices $\{M: m_{ij} \in [m_{ij}] \text { with } m_{ij} = m_{ji}\}$. For example, if $$M_1 = \begin{bmatrix} -3&0.1 \\ 0.1&-3 \end{bmatrix}$$ then $M_1 \in [M]$. However if $$M_2 = \begin{bmatrix} -3&0.2 \\ 0.1&-3 \end{bmatrix}$$ then $M_2 \notin [M]$.

Properties of scalar matrices, such as positive-definiteness and non-singularity are defined for interval matrices by requiring the property to hold for each scalar matrix belonging to the interval matrix. In this paper we are interested in symmetric interval matrices since we will calculate interval Hessian matrices over a given hyper-rectangular area, $[X]=[ [x_1],[x_2],\ldots ,[x_n]]^T$. Therefore we deal only with real eigenvalues.

### **Definition 2.1**

(*Positive definite interval matrix*) An interval matrix [*M*] is positive definite iff every $M \in [M]$ is positive definite.

### **Definition 2.2**

(*Non-singular interval matrix*) An interval matrix [*M*] is non-singular iff every $M \in [M]$ is non-singular.

For a $n \times n$ symmetric matrix *M* we denote with $\lambda _i(M)$ the *i*-th largest eigenvalue of *M*, with $\lambda _n(M) \le \lambda _{n-1}(M) \le \cdots \le \lambda _1(M)$. The eigenvalues of a symmetric interval matrix are defined as follows.

### **Definition 2.3**

(*Eigenvalues of an interval matrix*) The *i* *th* largest eigenvalue of a symmetric matrix [*M*] is defined as the set $\lambda _i([M]) = \{ \lambda _i(M): M \in [M] \}$.

### **Definition 2.4**

(*Index and coindex of scalar matrix*) The index (coindex), index(*M*) (coindex(*M*)), of a symmetric matrix *M* is the number of strictly negative (positive) eigenvalues of *M*.

### **Definition 2.5**

(*Index of symmetric interval matrix*) The index of a symmetric interval matrix [*M*] is defined as $\text {min} \{\text {index}(M): M \in [M]\}$.

Similarly we define the coindex for symmetric interval matrices.

### **Definition 2.6**

(*Inertia of a symmetric scalar matrix*) Given a symmetric matrix *M*, the inertia of *M*, *In*(*M*), is the triplet $( \pi (M) , \nu (M) , \delta (M) )$ of the numbers of positive, negative and zero eigenvalues of *M* respectively.

Note that $\pi$ and $\nu$ are the same as the index and coindex respectively.

### **Definition 2.7**

(*Inertia of a symmetric interval matrix*) Given a symmetric matrix [*M*], the inertia of [*M*], *In*([*M*]), is defined as $\text {min} \{\text {In}(M): M \in [M]\}$. That is, $\text {In}([M]) = \left( \underset{M \in [M]}{\text {min}}\pi (M) , \underset{M \in [M]}{\text {min}}\nu (M) , \underset{M \in [M]}{\text {min}}\delta (M) \right)$.

### **Definition 2.8**

(*Norm of an interval matrix*) We define the p-norm of an interval matrix, [*M*], as $\Vert [M]\Vert _p = \max \{ \Vert M\Vert _p : M \in [M] \}$.

It is easy to verify that by this definition all the conditions required to hold for a norm of a scalar matrix also hold for the norm of an interval matrix.

## Proposed approach

We use a branch-and-bound (B&B) algorithm and the formulation proposed in [29] (problem *P* below) in order to search for critical points:

P $$\begin{aligned} \begin{array}{lll} (P)&{}\underset{s,x}{\text {minimize}} &{}\quad s \\ &{}\text {subject to}&{}\quad \partial f(x) / \partial x_i - s \le 0 ,\quad i = 1, \ldots , n \\ &{}&{}\quad -\partial f(x) / \partial x_i - s \le 0 ,\quad i = 1, \ldots , n \\ &{}&{}\quad x_i \in [ \underline{x_i} , \overline{x_i} ] ,\quad i = 1, \ldots , n. \end{array} \end{aligned}$$

**Fig. 1:**
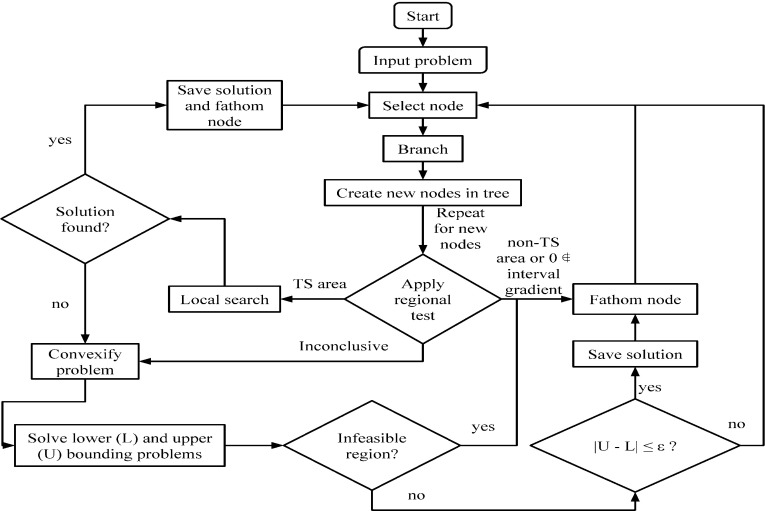
Algorithm flowchart

However, aiming to focus the computational effort on the location of TSs, we introduce a number of tests which can be used to bound the number of negative and positive eigenvalues of an interval matrix. In a branch-and-bound algorithm, at any given iteration, valid lower and upper bounds on the global minimum are calculated over hyper-rectangular subsets *R* of the initial domain *B*. By dividing each subset area improving lower and upper bounds are obtained. Whenever the lower bound of a given area is found to be greater than the best upper bound so far, the area is fathomed. We modify the approach by applying, prior to each bounding step, a test on the interval Hessian matrix, $[\nabla ^2f(R)]$, calculated over *R* by the natural interval extension [11] of the second derivatives $\partial ^2f / \partial x_i \partial x_j$. The interval Hessian can be seen as a superset of $\{ \nabla ^2f(x): x \in R \}$. If the test reveals that every matrix in $[\nabla ^2f(R)]$ has index $>$ 1 then we fathom the area *R*. If the test reveals that every matrix in $[\nabla ^2f(R)]$ is index-1 and coindex-$n-1$ then we can choose to perform a local search, since it can be shown (cf. Sect. 5) that this implies that there can be at most one TS in *R*. If a TS is found during the local search, we fathom the area. Otherwise the test is inconclusive and we proceed to the next step of the modified B&B algorithm. A flowchart of the proposed procedure is given in Fig. 1. A check to determine if zero is contained in the interval gradient is also applied at every iteration; if it is not the area is discarded.

## Regional tests

In this section, we introduce five regional tests related to the presence of TSs. The tests can be used to identify regions that do not contain any TS, or regions that contain at most one TS. The computational complexity of each test is reported in each case. If the tests are embedded within a branch-and-bound algorithm for the solution of Problem (P), the computational complexity of the solution of the convex lower bounding problem, which is NP-hard, dominates the overall cost. Furthermore, if the $\alpha$BB algorithm [2, 3] is used, the interval Hessian matrix information required in the tests is readily available from the construction of the lower bounding problem and an efficient implementation can be developed with minimal effort devoted to the application of tests. Examples of the application of each test can be found in the “Appendix”.

### The Gerschgorin test

We begin by developing a regional test based on the well-known theorem by Gerschgorin [30].

#### **Theorem 4.1**

(Gerschgorin) Given a matrix $M \in \mathbb {C}^{n\times n}$, define the radii $r_i = \sum _{i\ne j}|m_{ij}|$ and the discs $D_i(M) = \{z\in \mathbb {C}: |z-m_{ii}|\le r_i\}$. Then all the eigenvalues of *M* belong to the union $G(M)=\cup D_i(M)$. Furthermore, if the union of *k* of the discs $D_i(M)$ forms a disjoint set from the rest $n-k$ discs, then it contains exactly k eigenvalues.

An interval extension for the first part of the above theorem was given in [2] and used for the calculation of lower bounds for the eigenvalues of symmetric interval matrices. Here we are interested in the second part of Gerschgorin’s theorem, on counting the eigenvalues in disjoint sets. The extension in [2] is also valid for the second part of the theorem.

#### **Theorem 4.2**

(Interval extension) Given a $n \times n$ symmetric interval matrix [*M*], define the radii $r_i([M]) = \sum _{j=1}^n \text {max} \{ \underline{|m_{ij}|} , \overline{|m_{ij}|} \}$ and the intervals $D_i([M]) = [ \underline{m_{ii}} - r_i([M]) , \overline{m_{ii}} + r_i([M]) ]$ for $i=1,2,\ldots n$. Then all the eigenvalues of every $M \in [M]$ belong to the union $G([M])=\bigcup _i D_i([M])$. Furthermore, if the union of *k* of the intervals $D_i([M])$ forms a disjoint set from the other $n-k$ intervals, then it contains exactly k eigenvalues of every $M \in [M]$.

#### *Proof*

Based on the definition of the intervals $D_i([M])$, we have that $\forall M \in [M]$, $D_i(M) \subseteq D_i([M])$ for $i=1,2,\ldots ,n$. Thus $\forall M \in [M]$, $G(M) \subseteq G([M])$ $\Rightarrow$ $\forall M \in [M]$, $\sigma (M) \in G([M])$ where $\sigma (M)$ is the spectrum of *M*. To prove the second part of the theorem, assume, without loss of generality, that the union $U_k=\bigcup _{i=1}^k D_i([M])$, for some $k \in \{1,2,\ldots ,n\}$, is disjoint from $U_{n-k}=\bigcup _{i=k+1}^n D_i([M])$. Then, $\forall M \in [M]$, $\bigcup _{i=1}^k D_i(M) \subseteq U_k$ and $\bigcup _{i=k+1}^n D_i(M) \subseteq U_{n-k}$ and therefore by theorem 4.1 exactly *k* eigenvalues of *M* belong to $U_k$. $\square$

We give a pseudocode for a test based on Theorem 4.2, which we call the Gerschgorin test, in Algorithm 1. Regions for which the interval Hessian contains no negative disks (convex areas), or where a set of more than one discs lie on the negative side and are disjoint from the rest, are removed (lines 16–17 and 21–29 in Algorithm 1). By “discs” here we mean the intervals $D_i([M])$. Regions with one negative eigenvalue and all the other positive may also be identified (lines 18–19). Notice that the Gerschgorin test may be inconclusive even for a scalar matrix.
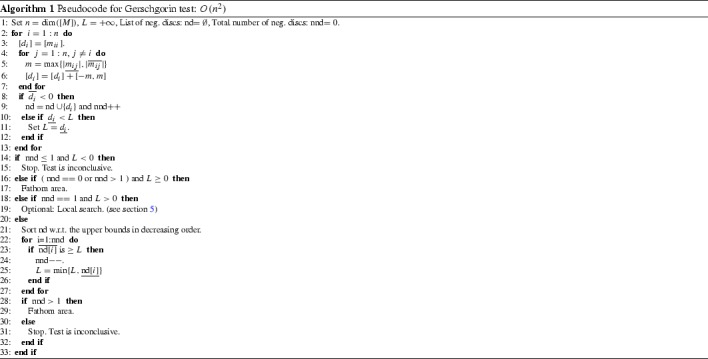


### Recursive inertia (RecIn) test

Based on Haynsworth’s theorem [5, 12] we can construct algorithms for obtaining bounds on the number of negative and positive eigenvalues of interval matrices.

#### **Theorem 4.3**

(Haynsworth) Given a symmetric matrix *M* partitioned in the form, $$M = \begin{bmatrix} A&B \\ B^T&C \\ \end{bmatrix}$$ and assuming *A* is non-singular, then, $\text {In}(M) = \text {In}(A)+\text {In}(C-B^TA^{-1}B)$.

Haynsworth’s Theorem can be extended in the interval case as follows:

#### **Theorem 4.4**

(Interval extension) Given a symmetric interval matrix [*M*] partitioned in the form, $$[M] = \begin{bmatrix} [A]&[B] \\ [B]^T&[C] \\ \end{bmatrix}$$ and assuming [*A*] is non-singular, then, $\text {In}([M]) \ge \text {In}([A])+\text {In}([C]-[B]^T[A]^{-1}[B])$.

Before we proceed with the proof we note that when we multiply two interval matrices, [*A*] and [*B*], we have that $[C]=[A][B] \supseteq \{ AB: A \in [A] \text { and } B \in [B] \}$. The proof of Theorem 4.4 is straightforward:

#### *Proof*

Let $[S] = [C]-[B]^T[A]^{-1}[B]$ and $S_x = \{ C-B^TA^{-1}B: A \in [A], B \in [B], C \in [C] \}$ with $[S] \supseteq S_x$. Then

1 $$\begin{aligned} \text {In}([M])= & {} \underset{A\in [A],B\in [B],C\in [C]}{\text {min}} \text {In}(A) + \text {In}(C-B^TA^{-1}B) \end{aligned}$$

2 $$\begin{aligned}\ge & {} \underset{A\in [A]}{\text {min}}\text {In}(A) + \underset{S\in S_x}{\text {min}} \text {In}(S) \end{aligned}$$

3 $$\begin{aligned}\ge & {} \underset{A\in [A]}{\text {min}}\text {In}(A) + \underset{S\in [S]}{\text {min}} \text {In}(S) \text {} \text { }=\text { } \text {In}([A]) + \text {In}([S]). \end{aligned}$$

$$\square $$

We can make use of Haynsworth’s theorem recursively, as shown by Cottle [7]. Cottle considers scalar matrices and chooses *A* to be a single non-zero entry in the diagonal. By interchanging corresponding rows and columns simultaneously, thus not affecting the eigenvalues, we bring the selected entry *A* to the top left position of the matrix. We note the sign of *A*, we then calculate $C-B^TA^{-1}B$ (the Schur complement of *A* in *M*), and repeat. If all the elements in the diagonal are zero, we are either left with a zero matrix or we can choose *A* to be of the form $$\begin{bmatrix} 0&a \\ a&0 \\ \end{bmatrix}$$. In this way, we can always calculate the complete inertia of a scalar matrix.

A straightforward adaptation of this recursive scheme for interval matrices [*M*] is simply to scan the diagonal for an interval that does not contain zero and re-arrange [*M*] as appropriate, calculate the interval Schur complement $[C]-[B]^T[A]^{-1}[B]$ and repeat. We should give priority to negative intervals. If at any point all the diagonal interval elements contain zero, then we cannot proceed further with the analysis and stop. Note that in the interval case, each time we find a negative (resp. positive) interval in the diagonal of a subsequent Schur complement, this means that all the scalar matrices contained in the initial interval matrix have a further negative (resp. positive) eigenvalue. In a similar manner, Meyer and Swartz [21] used Schur’s formula, $det(M) = det(A) det(C-B^TA^{-1}B)$, for a convexity test applied to interval matrices (such a test was mentioned in [7] for scalar matrices) along with a branch-and-bound method. In Algorithm 2 we give a pseudocode for the proposed recursive inertia test, RecIn.
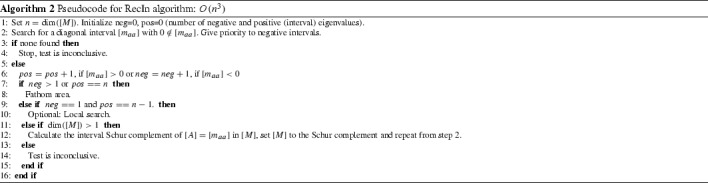


### Extended RecIn test

The RecIn test cannot proceed if all diagonal elements of the initial input matrix or of a subsequent Schur complement contain zero. We extend the RecIn algorithm to overcome this issue.

The following Lemma was given in [17].

#### **Lemma 4.5**

Given a $n \times n$ symmetric interval matrix [*M*] define the symmetric interval matrices

4 $$\begin{aligned}{}[L] = \{ l_{ii} = \underline{m_{ii}} \text { and } [l_{ij}] = [m_{ij}] \quad \text {for } i \ne j \} \end{aligned}$$

and

5 $$\begin{aligned}{}[U] = \{ u_{ii} = \overline{m_{ii}} \text { and } [u_{ij}] = [m_{ij}] \quad \text {for } i \ne j \}. \end{aligned}$$

Then $\forall M \in [M]$, there are $L \in [L]$ and $U \in [U]$ such that,

6 $$\begin{aligned} \lambda _i(L) \le \lambda _i(M) \le \lambda _i(U) \quad \text { for } i=1,2,\ldots ,n. \end{aligned}$$

#### **Corollary 4.6**

Given a $n \times n$ symmetric interval matrix [*M*] and defining the matrices [*L*] and [*U*] as above, then $\forall M \in [M]$,

7 $$\begin{aligned} \underset{U \in [U]}{\min }\nu (U) \le \text { } \nu (M) \text { } \le \text { } n-\underset{L \in [L]}{\min }\pi (L). \end{aligned}$$

#### *Proof*

Lemma 4.5 implies that $\forall M \in [M]$, there are $L \in [L]$ and $U \in [U]$ such that

8 $$\begin{aligned} \nu (U) \le \nu (M) \le \nu (L). \end{aligned}$$

Therefore $\forall M \in [M]$ we have,

9 $$\begin{aligned} \underset{U \in [U]}{\min }\nu (U) \text { } \le \text { } \nu (M) \text { } \le \text { } \underset{L \in [L]}{\max }\nu (L). \end{aligned}$$

Also, $n-\underset{L \in [L]}{\min }\pi (L) \ge \underset{L \in [L]}{\max }\nu (L)$ (the inequality stems from the fact that the matrix might have zero eigenvalues) and hence finally,

10 $$\begin{aligned} \underset{U \in [U]}{\min }\nu (U) \le \text { } \nu (M) \text { } \le \text { } n-\underset{L \in [L]}{\min }\pi (L). \end{aligned}$$

$$\square $$

In a similar way we can show that,

11 $$\begin{aligned} \underset{L \in [L]}{\min }\pi (L) \le \text { } \pi (M) \text { } \le \text { } n-\underset{U \in [U]}{\min }\nu (U), \forall M \in [M]. \end{aligned}$$

Based on Corollary 4.6, we introduce algorithms RecIn_U and RecIn_L. RecIn_U makes use of the [*U*] part of the initial input matrix [*M*] and of each subsequent Schur complement and is used to calculate a lower bound of $\underset{U \in [U]}{\min }\nu (U)$. In analogy, RecIn_L makes use of the [*L*] part and is used to calculate a lower bound of $\underset{L \in [L]}{\min }\pi (L)$. Thus, by (10) and (11), we obtain bounds for $\nu ([M])$ and $\pi ([M])$. We give the pseudocode for the RecIn_U in Algorithm 3 and then the extended recursive inertia test, xRecIn in Algorithm 4. We omit the pseudocode for RecIn_L since it is easy to derive it from RecIn_U.
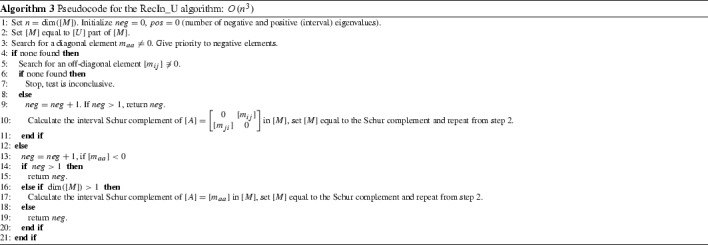


Note that for the calculation of the Schur complement in step 10 of the RecIn_U algorithm, the inverse of [*A*] is simply $$[A]^{-1}=\begin{bmatrix} 0&1/[m_{ij}] \\ 1/[m_{ji}]&0 \\ \end{bmatrix}$$.
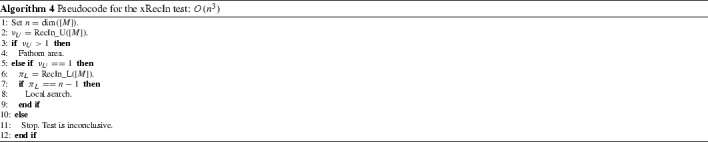


### $2\times 2$ Inertia test

Another possible way to make use of Theorem 4.3 for our purpose is to choose [*A*], in $$[M] = \begin{bmatrix} [A]&[B] \\ [B]^T&[C] \\ \end{bmatrix}$$, to be any of the $2\times 2$ diagonal sub-matrices of [*M*], $$[A_{ij}] = \begin{bmatrix} [m_{ii}]&[m_{ij}] \\ [m_{ji}]&[m_{jj}] \\ \end{bmatrix}$$. The maximum eigenvalue, $\overline{\lambda _{ij}} = \underset{A_{ij} \in [A_{ij}]}{\max } \lambda _1(A_{ij})$, of each of these matrices is

12 $$\begin{aligned} \overline{\lambda _{ij}} =\frac{ \overline{m_{ii}} + \overline{m_{jj}} + \sqrt{(\overline{m_{ii}} - \overline{m_{jj}})^2 +4\max \{\underline{m_{ij}}^2,\overline{m_{ij}}^2\}} }{2}. \end{aligned}$$

If $\overline{\lambda _{ij}} < 0$ for any of the sub-matrices then by Theorem 4.3 we know that every $M \in [M]$ has at least two negative eigenvalues and thus we can fathom the corresponding area. In Algorithm 5 we give a pseudocode for this test to which we refer as the $2\times 2$ inertia test.
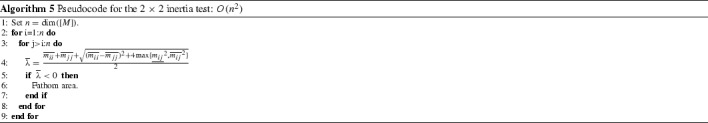


Note that the $2\times 2$ inertia test does not remove TSs and minima and that it may be inconclusive even for a scalar matrix. However, it is computationally cheap and it is easy to implement. Furthermore, it is straightforward to show that this test is more effective than the Gerschgorin test in identifying non-TS areas. More formally we have the following:

#### **Proposition 4.7**

Given a $n \times n$ symmetric interval matrix [*M*], if the Gerschgorin test reveals that index $([M])>1$ then so does the $2\times 2$ inertia test. The reverse is not always true.

#### *Proof*

Since the Gerschgorin test reveals that index$([M])>1$, this implies that there are at least two rows of [*M*], *i* and *j*, for which

$$\begin{aligned} \overline{m_{ii}} + \sum _{k=1,k\ne i}^{n} \max \{|\underline{m_{ik}}| , |\overline{m_{ik}}| \} < 0 \text { and } \overline{m_{jj}} + \sum _{k=1,k\ne j}^{n} \max \{|\underline{m_{jk}}| , |\overline{m_{jk}}| \} < 0. \end{aligned}$$

This implies that

13 $$\begin{aligned} \overline{m_{ii}} +\max \{|\underline{m_{ij}}| , |\overline{m_{ij}}| \} < 0 \text { and } \overline{m_{jj}} +\max \{|\underline{m_{ji}}| , |\overline{m_{ji}}| \} < 0. \end{aligned}$$

From (13) and Theorem 4.2 we have that for $$[M_{ij}]=\begin{bmatrix} [m_{ii}]&[m_{ij}] \\ [m_{ji}]&[m_{jj}] \\ \end{bmatrix}$$, $\overline{\lambda }([M_{ij}]) < 0$ and since the $2\times 2$ inertia test provides the exact upper bound of $\lambda ([M_{ij}])$, it also reveals that index$([M])>1$. $\square$

Finding a counter-example to show that the reverse is not always true is easy (see “$2 \times 2$ inertia test example” in the Appendix).

### Rohn test

The last test we present is based on Rohn’s method [17] which is derived from the interval extension of Weyl’s inequality [10].

#### **Theorem 4.8**

(Weyl) Given $n\times n$ symmetric (scalar) matrices *C* and *E*, then

14 $$\begin{aligned} \lambda _k(C) + \lambda _n(E)\text { } \le \text { } \lambda _k(C+E) \text { } \le \text { }\lambda _k(C) + \lambda _1(E), \quad \text {for } k=1,2,\ldots ,n. \end{aligned}$$

$\text {where for any matrix} M, \lambda _n(M) \le \cdots \le \lambda _1(M)$. Any given interval matrix [*M*] can be written as $C+[E]$ where $c_{ij} = (\overline{m_{ij}} + \underline{m_{ij}}) / 2$ and $[e_{ij}]=[c_{ij} - \underline{m_{ij}} , \overline{m_{ij}} - c_{ij} ]$. Calculating lower and upper bounds, $\underline{\lambda _n}$ and $\overline{\lambda _1}$, for $\lambda _n([E]) = \{ \lambda _n(E): E \in [E] \}$ and $\lambda _1([E]) = \{ \lambda _1(E): E \in [E] \}$ respectively, leads to the theorem by Rohn:

#### **Theorem 4.9**

(Rohn) Given a symmetric interval matrix [*M*] = $C+[E]$, then

15 $$\begin{aligned} \lambda _k(C) + \underline{\lambda _n} \text { } \le \text { } \lambda _k(C+[E]) \text { } \le \text { }\lambda _k(C) + \overline{\lambda _1}, \quad \text {for } k=1,2,\ldots ,n. \end{aligned}$$

Note that because *C* has been defined as the center matrix of [*M*], $\underline{\lambda _n} = -\overline{\lambda _1}$ and also that the widths of the intervals $\lambda _k([M])$ are all the same. We can calculate $\underline{\lambda _n}$ (and $\overline{\lambda _1}$) using a number of methods (see [2, 27]), the simplest being the interval extension of Gerschgorin’s theorem ($O(n^2)$) and the most expensive being the Hertz–Rohn method ($O(2^{n-1})$) [15, 16, 26]. The Rohn test is summarized in Algorithm 6.
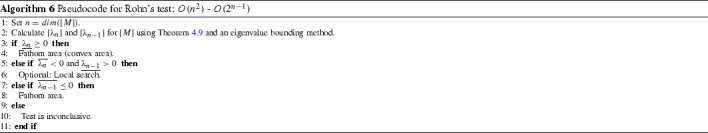


## Index-1 areas

In Sect. 3 we stated that hyper-rectangular areas where every matrix is index-1 and coindex-$n-1$ has at most one TS. We give a proof of this statement here. The proof is straightforward and we state it for completeness.

### **Theorem 5.1**

Assume we have a function $f \in C^2$, $f:B \subseteq \mathbb {R}^n \rightarrow \mathbb {R}$ where *B* is an open hyper-rectangular box. If $\nabla ^2f(x)$ is index 1 and coindex $n-1$ $\forall x \in B$ then there is at most one TS in *B*.

### *Proof*

If *f* has any critical points in *B* then by the assumption that $\nabla ^2f(x), x \in B$ is index-1 and coindex-$n-1$, they would be TSs. Now assume that $x_1,x_2 \in B$ with $x_1 \ne x_2$ are critical points of *f*. Then, by the mean value theorem

16 $$\begin{aligned} \nabla f(x_2) = \nabla f(x_1) + \nabla ^2 f(\xi )(x_2-x_1), \end{aligned}$$

for some $\xi$ between $x_1$ and $x_2$ and since *B* is a hyper-rectangle $\Rightarrow$ $\xi \in B$. However, $\nabla f(x_1)=\nabla f(x_2)=0$ and therefore

17 $$\begin{aligned} \nabla ^2 f(\xi )(x_2-x_1)=0 \Rightarrow \nabla ^2 f(\xi ) \text { singular }, \end{aligned}$$

which contradicts our assumption. $\square$

In practice the interval Hessian, $[\nabla ^2f(B)]$, over *B* would be an overestimation of $\{ \nabla ^2f(x): x \in B \}$. Hence, if the assumptions of Theorem 5.1 are true for $[\nabla ^2f(B)]$, they are also true for $\{ \nabla ^2f(x): x \in B \}$. At such cases we can perform a local search using Newton’s method for the unique critical point and if we locate a solution we can save this solution and fathom the corresponding area.

## Completeness of the tests

The proposed tests take as input a symmetric interval matrix [*M*] and aim to verify if $\exists M \in [M]$ such that, index$(M)=1$ and coindex$(M)=n-1$. Moreover, we might, optionally, try to verify if $\forall M \in [M]$, index$(M)=1$ and coindex$(M)=n-1$. The $2\times 2$ inertia test is an exception since it attempts to verify if index$(M) > 1 \text { } \forall M \in [M]$. In any case a test might fail to provide a definitive answer and thus be inconclusive. By considering under what circumstances a test may be inconclusive, we can classify the proposed tests using the following definitions.

### **Definition 6.1**

(*Complete test*) A test is called complete if it is never inconclusive.

### **Definition 6.2**

($\epsilon$-*complete test*) A test is called $\epsilon$-complete if $\forall$ $n\times n$ nonsingular, scalar matrix *C*, $\exists$ $\epsilon > 0$ such that $\forall$ [*E*] with $\Vert [E]\Vert <\epsilon$ the test is not inconclusive for $C+[E]$ as input.

### **Definition 6.3**

(*Incomplete test*) A test is called incomplete if it is not $\epsilon$-complete.

We note that in the above definitions, for any test, we assume infinite-precision arithmetic and also that we know the maximum number of steps a priori.

The Gerschgorin and $2\times 2$ inertia tests are incomplete since they can be inconclusive even for scalar matrices. The recursive inertia test is also incomplete since it cannot deal with matrices where all the diagonal elements contain zero. The extended recursive inertia test and Rohn test are $\epsilon$-complete. We do not know of any method that can result in a complete test or if a complete test is even possible. In the next section we prove that this is an NP-hard problem.

We could attempt to construct a complete test with the following reasoning. The Hertz–Rohn method [15] gives the exact lower and upper bounds of the smallest and largest eigenvalue, respectively, of any symmetric interval matrix [*M*]. It does so by calculating the smallest and largest eigenvalues over a finite number ($2^{n-1}$) of scalar matrices $M \in [M]$. The entries of these scalar matrices are either $\underline{m_{ij}}$ or $\overline{m_{ij}}$. Based on this, we might ask whether it is possible to have an a priori way of identifying a finite number of matrices in any given symmetric interval matrix [*M*], so that we can find the exact lower bound of index ([*M*]). We can show that, unlike the case of calculating the extreme eigenvalues, this is not possible if each element $m_{ij}$ is chosen as a function only of $[m_{ij}]$. This is expressed more formally in the following proposition.

### **Proposition 6.4**

Define a set $S=\{S_1,S_2,\ldots ,S_s\}$ where each $S_k$, $k=1,2,\ldots ,s$, is a set of functions $m_{ij}^{(k)}:\mathbb {R}^2\rightarrow \mathbb {R}$ for $i,j=1,2,\ldots ,n$ with $i\le j$ such that $l \le m_{ij}^{(k)}(l,u) \le u$ for any $l,u \in \mathbb {R}$ with $l\le u$. Given a $n\times n$ symmetric interval matrix [*M*], the set *S* defines a set, *S*([*M*]), of scalar matrices $M_1,M_2,\ldots ,M_s \in [M]$.

For any choice of *S* there is always a matrix [*M*] for which the set *S*([*M*]) fails to identify correctly the lower bound of index([*M*]). That is, $\exists M^* \in [M]$ such that index $(M^*) < \min \{\text {index}(M): M \in S(M) \}$.

### *Proof*

Consider a matrix of the form $$[M]=\begin{bmatrix} 1&1&b \\ 1&2&[c] \\ b&[c]&d_3 \\ \end{bmatrix}$$. From Theorem 4.3, $\forall M \in [M]$ we have

18 $$\begin{aligned} In(M) = In(1) + In(1) + In(-c^2+2cb-2b^2+d_3) \text { with } c \in [c]. \end{aligned}$$

The roots of $h(c) = -c^2+2cb-2b^2+d_3$ are given by $c_1^*,c_2^* = b \pm \sqrt{d_3-b^2}$. The distance between the roots is $d(c_1^*,c_2^*)=2\sqrt{d_3-b^2}$ and the midpoint is *b*. The function *h* is concave and thus positive in $(c_1^*,c_2^*)$ and negative outside of $[c_1^*,c_2^*]$.

For a given set *S*, $|S|=s$ the scalar matrices $M_1,M_2,\ldots ,M_s \in S([M])$ will have a corresponding entry $c_1,c_2,\ldots ,c_s \in [c]$. By appropriately choosing values for *b* and $d_3$, for example, $b=(c_k+c_{k+1})/2$ and $b^2 < d_3 < d(c_k,c_{k+1})^2/4 + b^2$ (such that $0 < 2\sqrt{d_3-b^2} < d(c_k,c_{k+1})$), we would have that $\forall M_i \in S([M])$, index$(M_i)=1$. However, the matrix $M^* \in [M]$ with $c=b$ would have index$(M^*)=0$.$\square$

### **Corollary 6.5**

There is no choice of *S* such that for any $n\times n$ symmetric interval matrix [*M*], *S*([*M*]) provides correct bounds for $\lambda _i([M])$, $i=1,2,\ldots ,n$.

### *Proof*

If such a choice of *S* existed then it would also allow the correct calculation of the bounds for the index of any symmetric interval matrix, which contradicts Proposition 6.4.

$$\square $$

A summary with the characteristics of each test is given in Table 1.

**Table 1:** Summary of the tests

Test	Completeness	Complexity	Comments
Gerschgorin	Incomplete	$O(n^2)$	Effective when diagonal entries are large with respect to off diagonal
$2\times 2$ Inertia	Incomplete	$O(n^2)$	Does not remove minima. Simple to implement
Rohn	$\epsilon$-complete	$O(n^2)-O(2^{n-1})$	Requires direct calculation of eigenvalues
RecIn	Incomplete	$O(n^3)$	Not applicable when all diagonal entries contain zero
xRecIn	$\epsilon$-complete	$O(n^3)$	Can handle cases where all diagonal entries contain zero

## Algorithmic complexity

In this section we investigate the algorithmic complexity of the problems that we aim to solve with the algorithms given in Sect. 4 that is, identifying a TS matrix or a non-TS matrix. By TS and non-TS we mean, given a symmetric interval matrix [*M*], identifying if $\forall M \in [M]$, index$(M)=1$ and coindex(M)$=n-1$ or if $\not \exists M \in [M]$ with index$(M)=1$ and coindex(M)$=n-1$ respectively. Rohn [25] proved that checking positive definiteness of an interval matrix is an NP-hard problem.

### **Theorem 7.1**

The decision problem:

- Instance: A $n\times n$ symmetric interval matrix [*M*].
- Question: Is [*M*] positive definite?

is NP-hard.

The problem of positive definiteness can be trivially reduced in polynomial time to the following problem.

### **Corollary 7.2**

The decision problem:

- Instance: A $n\times n$ symmetric interval matrix [*M*] and integer $k\in \{1,2,\ldots ,n\}$
- Question: Is index$([M]) = k$ and coindex$([M])=n-k$ ?

is NP-hard.

### *Proof*

Simply consider the block interval matrix

19 $$\begin{aligned}{}[M] = \begin{bmatrix} D&0 \\ 0&[A] \end{bmatrix} \end{aligned}$$

where *D* can be any diagonal $k \times k$ matrix with all the diagonal entries being negative and [*A*] a symmetric interval matrix. Checking if index$([M])=k$ and coindex$([M])=n-k$ is equivalent to checking if [*A*] is positive definite. $\square$

Therefore identifying a TS matrix is NP-hard. With the help of Haynsworth’s theorem and using the same reduction as in [23], used for proving that checking the positive semi-definiteness of an interval matrix is NP-hard, we can prove the NP-hardness of identifying a non-TS matrix. First we give the following lemma from [23].

### **Lemma 7.3**

The decision problem:

- Instance: A positive integer *m* and an *m*-dimensional vector $a, \Vert a\Vert _2 \le 0.1$ with rational positive entries.
- Question: Determine whether $\max \{z^T(I_m-aa^T)z: z \in \mathbb {R}^m, \Vert z\Vert \le 1 \} \le m - 1/d^2(a)$ where *d*(*a*) is the smallest common denominator of the entries of *a*.

is NP-complete.

### **Theorem 7.4**

The decision problem:

- Instance: A $n\times n$ symmetric interval matrix [M].
- Question: $$\exists M \in [M] \text { with } \text {index}(M)=1 \text { and } \text {coindex}([M])=n-k$$?

is NP-hard.

### *Proof*

Given integer *m* and vector *a*, set $A=(I_m-aa^T)^{-1}$, $\mu = m-1/d^2(a)$ and define the matrix

20 $$\begin{aligned}{}[M] = \begin{bmatrix} A&[z] \\ [z]^T&\mu \end{bmatrix}, \text { } [z]=[-1,1]. \end{aligned}$$

Note that $I_m-aa^T$ is positive definite and thus *A* exists and is also positive definite. From Theorem 4.3, we have that $\forall M \in [M]$,

21 $$\begin{aligned} In(M) = In(A) + In \left( \mu -z^T (I_m-aa^T) z \right) \end{aligned}$$

Since $$In(A)=(m,0,0)$$, [*M*] contains an index-1 matrix iff $$ \exists $$ $$z^* $$ such that $$ \mu -z^{^*T} (I_m-aa^T) z^* < 0 $$ which would imply a “no” answer to problem 7.3. $$\square $$

## Results

The proposed tests have been implemented in the $\alpha$BB algorithm [1]. The use of the $\alpha$BB algorithm for solving problem (P) requires the calculation of the second derivatives of the constraints, which include first derivatives of the function *f*. Therefore, function *f* must be three-times continuously differentiable in the specific implementation we have developed. The tests presented here, however, are applicable to $C^2$ functions and can readily be integrated within algorithms that do not require the constraints to be in $C^2$, e.g. [18]. As mentioned previously, an efficient implementation of the tests can be constructed by using the interval values of the second-order derivatives of *f* that can be computed when calculating $\alpha$ values for the underestimators. A more basic implementation has been used here, so that the computational performance provides a worst-case analysis of the cost of the tests.

**Table 2:** CPU times and number of solutions of each type found for each run for the Ackley function

Test	CPU time (s)	CPU time with local search (s)	#Mins	#TSs	#Other solutions
No test	64	–	27	81	84
Gersch.	38	32	0	81	11
$2\times 2$ Inertia	33	–	27	81	0
Rohn	30	21	0	81	0
RecIn	28	19	0	81	0

We investigate the performance of the proposed tests on a number of problems. For each problem we perform one run using no test and separate runs using each test without local search. For the Gerschgorin, RecIn and Rohn tests we also perform runs with local search in order to evaluate whether there would be any improvement regarding the CPU time. For bounding the eigenvalues in Rohn’s test we used the interval extension of Gerschgorin’s theorem [2]. For each problem we give a table containing the CPU times for each run and the corresponding number of (non-degenerate) minima, TSs and other solutions found and a graph which shows the number of unfathomed nodes at each iteration for each run. We also give a summary of the success rates (No. of nodes fathomed by test/No. of times test applied) for each test in each problem (no local search applied). The computations were performed on an Intel CPU @ 3060 MHz using an absolute convergence tolerance of $10^{-6}$ and a minimum box size of $10^{-6}$.

### Problem 1: Ackley’s function

**Fig. 2:**
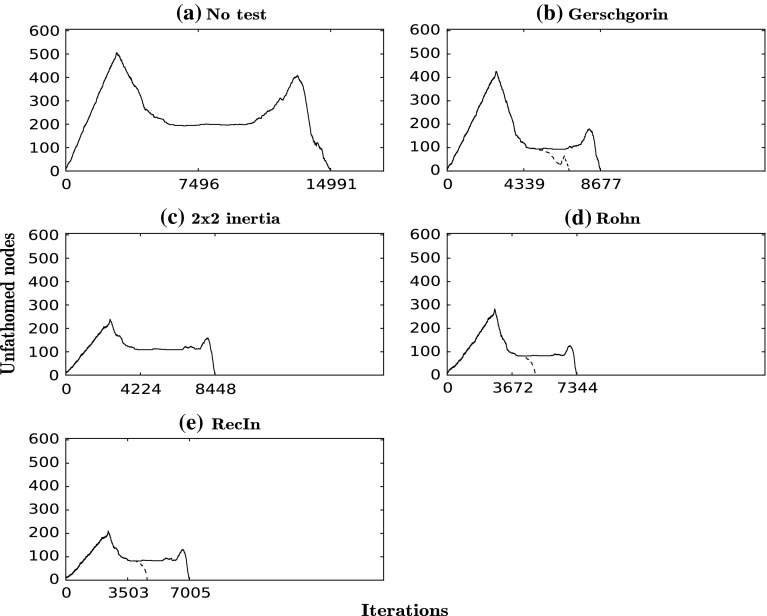
Number of unfathomed nodes at each iteration for each run for the Ackley function. *Dashed curves* correspond to the same test but with local search

For the first example, we apply the algorithm to Ackley’s function:

$$\begin{aligned} {f(x) = -20\exp \left( -0.2\sqrt{\frac{1}{n} \sum _{i=1}^n x_i^2}\right) - \exp \left( \frac{1}{n}\sum _{i=1}^n \cos (2\pi x_i)\right) +20+e}, \end{aligned}$$

with $n=3$ and $x \in [0.5,3]^3$. This low-dimensional example has 81 first-order saddle points, which are found with all configurations of the algorithm (with or without tests). We can observe from Table 2 that, with the application of the regional tests, the CPU time can be reduced by more than 50 % in comparison to the “no test” case (location of all critical points), which has a CPU time of 64 s. A further reduction in CPU time of 15–30 % is achieved with the application of the local search over areas that are found to have index-1. The RecIn test has the best performance, with a CPU time of only 19 s when the local search is also applied, with the Rohn test also exhibiting very strong performance. Furthermore, the Rohn and RecIn tests only return the TSs as solutions while the Gerschgorin test and the $2 \times 2$ test return a number of non-TS critical points too: 11 of the 84 higher-order saddle points or maxima in the case of the Gerschgorin test and all 27 minima in the case of the $2\times 2$ test. In Fig. 2, the number of open nodes in the branch-and-bound tree is reported as a function of iteration number for every test. The scales used in the five panels are the same to make comparison easier. The significant reduction in the number of iterations when the tests are applied is evident and the branch-and-bound tree is found to be much smaller (Fig  2; Table 2).

**Fig. 3:**
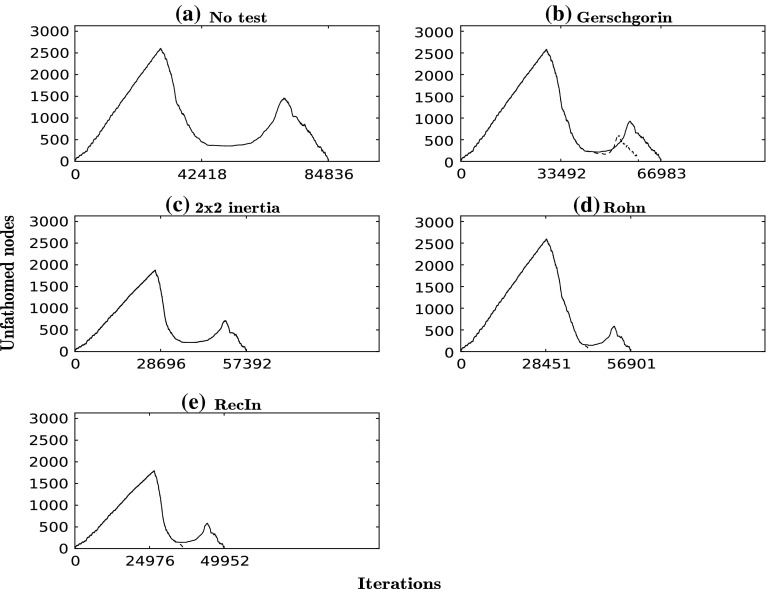
Number of unfathomed nodes at each iteration for each run for the Levy function. *Dashed curves* correspond to the same test but with local search

### Problem 2: Levy function

In this example we use a Levy function: $f(x) = \sin ^2(\pi y_1) + \sum _{i=1}^{n-1} (y_i-1)^2 [1+10\sin ^2(\pi y_{i+1})] + (y_n-1)^2$, where $y_i=1+(x_i-1)/4$. In our case, $n=5$ and $x\in [-5,5]^5$. This more challenging example has a total of 349 stationary points of which 142 are transition states and 63 are minima, as can be seen in Table 3. Notice that the Hessian of *f* is tridiagonal. Again, without local search, we see a significant reduction in CPU time, of between 9 and 38 % (Table 3), and in iteration number, of up to 41 % (Fig. 3). The maximum overall CPU time reduction achieved with the use of a test combined with local search is of 50 %. The RecIn test has the best performance with a CPU time of 108 s in contrast to the 218 s required when no regional test is applied. As in the first example, the Rohn test provides the second-best performance when accompanied by local search. However, without local search, the second-best performance is achieved with the $2 \times 2$ inertia test. Both Rohn and RecIn tests return only the TSs as solutions, whereas the $2 \times 2$ inertia test leads to the identification of all 63 minima and the Gerschgorin test to the identification of 58 other stationary points.

**Table 3:** CPU times and number of solutions of each type found for each run for the Levy function

Test	CPU time (s)	CPU time with local search (s)	#Mins	#TSs	#Other solutions
No test	218	–	63	142	144
Gersch.	197	174	0	142	58
$2\times 2$ Inertia	152	–	63	142	0
Rohn	169	140	0	142	0
RecIn	134	108	0	142	0

**Table 4:** CPU times and number of solutions of each type found for each run for the Himmelblau function

Test	CPU time (s)	CPU time with local search (s)	#Mins	#TSs	#Other solutions
No test	520	–	64	192	473
Gersch.	332	319	0	192	0
$2\times 2$ Inertia	272	–	64	192	0
Rohn	333	320	0	192	0
RecIn	248	237	0	192	0

**Fig. 4:**
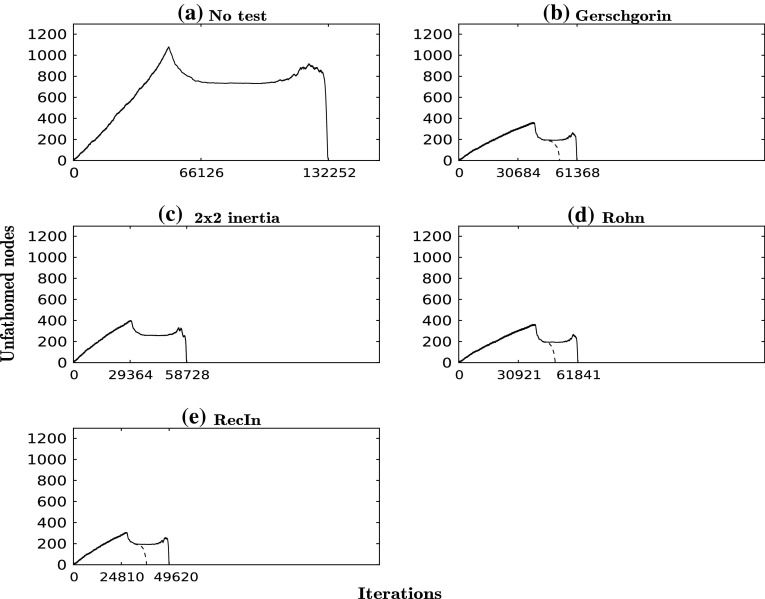
Number of unfathomed nodes at each iteration for each run for the Himmelblau function. *Dashed curves* correspond to the same test but with local search

### Problem 3: Himmelblau’s function

In this example we use an extension of Himmelblau’s function to multiple dimensions: $f(x) = \sum _{i<j}^n \left[ (x_i^2+x_j-11)^2+(x_i+x_j^2-7)^2 \right]$, where $n=6$ and $x \in [-5,5]^6$. The results are presented in Table 4 and Fig. 4. Although this example has only one variable more than Problem 2, the number of stationary points is much greater, with 729 points in total, of which 192 are transition states and 64 are minima. There is therefore a considerable computational cost to searching for all stationary points. The basic algorithm, without any regional tests, identifies all 729 points in 520 CPU seconds, compared to 218 CPU seconds in Problem 2. In contrast, the use of tests without local search leads to a reduction in CPU time of between 36 and 52 % and the use of tests with local search to a reduction of between 38 and 54 % overall. It is clear from these numbers that the application of one test provides most of the performance improvement in this example, and that the local search, albeit beneficial, has a modest impact on the overall CPU times. Once more the RecIn test is the most effective test, reducing the CPU time by a factor greater than 2 with respect to the case when no test is applied. In this particular case, the Gerschgorin test does not lead to the identification of additional stationary points. The $2 \times 2$ test offers second-best performance, and identifies all minima as well as all transition states.

### Problem 4: 2D-XY lattice model

For the last example we use the 2D-XY lattice model [19]:

$$\begin{aligned} H = \frac{1}{2} \sum _{k\in \varLambda } \sum _{l\in N(k)} [1-\cos ( \theta _k-\theta _l )] \end{aligned}$$

where $\varLambda = \{1,2,\ldots ,9\}$ and $N\{k\}$ is the set of indices of the neighbouring lattice points to the lattice point with index *k*.

**Fig. 5:**
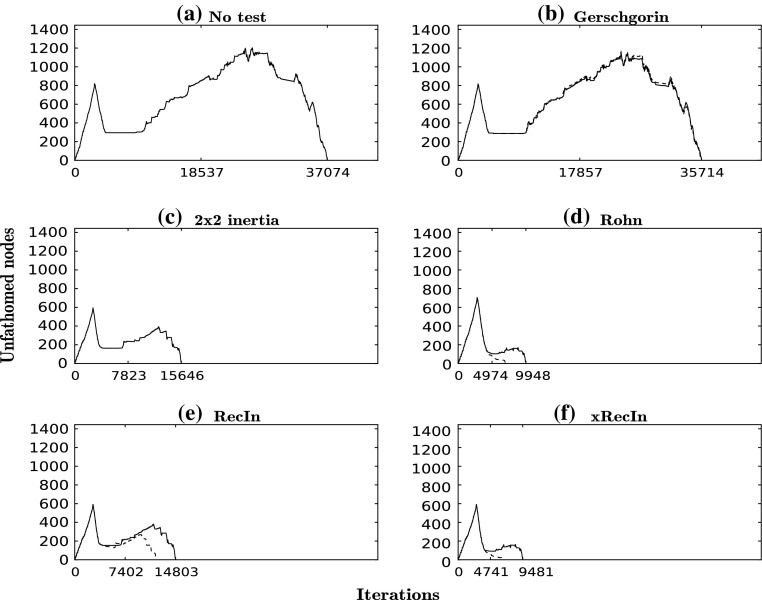
Number of unfathomed nodes at each iteration for each run for the 2D-XY lattice model function. *Dashed curves* correspond to the same test but with local search

The 2-dimensional XY lattice model has been studied, amongst others, in [19, 20]. The model exhibits exponential growth of the number of stationary points as the number of lattice points grows. Here, we consider a $3\times 3$ lattice where $\theta _7 = \theta _8 = \theta _9 = 0$, $\theta _3=\theta _6 = \pi /2$ and $\theta _i \in [-\pi ,\pi ]$ for $i=1,2,4,5$. Thus, this is a 4-dimensional problem. This example has a relatively small number of stationary points (33), with only 5 transition states and one minimum, and the algorithm without tests identifies all these points within 86 CPU seconds. However, the performance of the tests, as presented in Table 5 and Fig. 5, is more disparate than in previous examples. The frequent appearance of interval Hessian matrices where some or all diagonal elements include zero makes this example more challenging for some of the tests. Thus, the Gerschgorin test leads to a reduction in the total number of iterations of less than 4 %, and no reduction in the CPU time, which remains at 86 CPU seconds. This is due to the fact that some Gerschgorin discs overlap when zero is present in the diagonal elements and this may result in the test being inconclusive. We note that the computational cost could be reduced with a more efficient implementation that permits the re-use of the calculations of the interval Hessian matrix elements carried out while constructing the $\alpha$BB underestimators for the purpose of the test. Nevertheless, based on the implementation used here, the Gerschgorin test does not lead to a change in CPU time and identifies 26 “other” solutions in addition to the 5 transition states. Secondly, in this case the Rohn test performs better than the RecIn test: this latter test leads to a larger CPU time than the Rohn test and fails to remove a number of non-TS solutions. The reason for this is the presence of zeros in the diagonal entries of the interval Hessian matrices that prevent application of the RecIn test. However, the use of the xRecIn test can overcome this problem and, as can be seen in Table 5, it performs slightly better than the Rohn test.

**Table 5:** CPU times and number of solutions of each type found for each run for the 2D-XY lattice model function

Test	CPU time (s)	CPU time with local search (s)	#Mins	#TSs	#Other solutions
No test	86	–	1	5	27
Gersch.	86	86	0	5	26
$2\times 2$ Inertia	46	–	1	5	16
Rohn	33	28	0	5	0
RecIn	45	40	0	5	16
xRecIn	32	27	0	5	0

### Overall performance of the tests

Overall, the application of the proposed tests leads to a reduction in the number of iterations and this is usually accompanied by a significant reduction in CPU time, by up to 50 %. The application of the local search always leads to a reduction in both CPU time and iteration number. The most appropriate version of the recursive inertia test (RecIn or xRecIn) test, as indicated by the presence or not of zeros in the diagonal elements of the interval Hessian matrix, is found to provide the best performance in every case. The Rohn test usually performs well too, while the CPU time reduction is not as large with the Gerschgorin and $2 \times 2$ inertia tests. The worst performance was observed in applying the Gerschgorin test to Problem 4, where the presence of zeros in the Hessian matrix results in overlap of the Gerschgorin discs and the inability to eliminate most nodes. This provides a useful insight into the types of problems for which this test is most appropriate.

It is instructive to consider the success rates of the tests. In the proposed approach, the interval gradient test was applied at every node of the branch-and-bound tree and the chosen test was then applied at every node at which the interval gradient test was passed. The success rate of each test is calculated as the ratio of the number of nodes fathomed by a test to the number of times this test was applied, and is reported in Table 6. The success rates obtained are of the order of a few percent, with a maximum value of 5.35 %. As discussed, the lowest overall success rate is exhibited by the Gerschgorin test, while the RecIn test is most consistently successful. As can be expected, the tests tend to become more effective as the nodes become smaller for two reasons. First, in the test cases considered here, there are many stationary points and a large portion of the domain contains points at which the Hessian matrix is index-1 (whether they are index-1 critical points or not). Second, the larger the volume of the node the larger the overestimation inherent in the evaluation of the interval Hessian matrix, so that large nodes cannot be eliminated easily. Despite the relative inefficiency of the tests, the CPU-times for the problems presented are halved, indicating that the tests play a useful role. Further gains in CPU time may be derived by imposing a maximum threshold on the size of the node so that tests are only applied to “small-enough” nodes.

A strategy to reduce the number of iterations is to apply multiple tests. The RecIn/xRecIn tests generally lead to the elimination of regions that are eliminated by other tests. However, the reverse is not true. If the tests are applied in series, it is therefore advantageous to apply the least computationally demanding tests first, specifically Gerschgorin and $2 \times 2$ inertia and to follow this with RecIn/xRecIn tests. This strategy was deployed on the test problems, but due to the relatively low dimensionality of the examples (up to 6 variables), it did not lead to an improvement in CPU time compared to applying RecIn/xRecIn only. It would be interesting to explore this strategy further by deploying the tests in parallel on larger problems.

**Table 6:** Success rates in percentages for each test for each problem

Test/problem	Ackley	Levy	Himmelblau	2D-XY
Gersch.	1.93	0.37	1.40	0.02
$2\times 2$ Inertia	3.31	1.75	4.35	1.40
Rohn	4.06	0.73	1.23	2.79
RecIn	4.35	2.37	5.35	1.45
xRecIn	–	–	–	2.91

## Conclusions

In this paper we considered the problem of enclosing all transition states (TSs) of general nonlinear functions in $C^2$ using global deterministic methods. We introduced five tests that can be applied prior to the bounding step of branch-and-bound algorithm. These tests help to identify areas of the search space which do not contain any TSs or may contain at most one. In the first case we fathom/remove the area while in the second we perform a local search and if a solution is found we then fathom the area. With the tests we aim to focus the computational effort on the location of TSs rather than the identification of all critical points. We have implemented this approach within the $\alpha$BB algorithm and presented the successful application of the proposed tests to a number of low-dimensional problems in $C^3$, with up to six variables. The problems typically exhibit numerous stationary points. The results indicate that the addition of the tests can reduce the computational time significantly while locating all the transition states successfully. Furthermore, the use of a local search in areas that are identified to contain at most one TS is found to be advantageous, reducing both CPU time and iteration number. We note that the proposed tests can be used within any branch-and-bound algorithm or within the interval Newton method and that, with the exception of the $2\times 2$ inertia test, they can be altered in order to locate any index-k critical point. The RecIn/xRecIn tests are particularly effective for all problems considered. The use of the tests is a useful step towards the application of a branch-and-bound algorithm to the identification of transition states for larger problems: within the $\alpha$BB algorithm, the tests can be implemented at relatively low cost because the required interval Hessian matrix is computed implicitly as art of the underestimation procedure. Thus, the overhead arising from the tests can be kept low, while achieving a reduction in iteration number.

## Appendix

### Gerschgorin test example

Consider the matrix,

$$\begin{aligned}{}[M] = \begin{bmatrix} [-3,-2]&[-0.5,0.5]&[-1,1] \\ [-0.5,0.5]&[-4,-3]&[0.5,1] \\ [-1,1]&[0.5,1]&[2.5,3] \\ \end{bmatrix}. \end{aligned}$$

Then we have that $r_1 = r_2 = r_3 = 1.5$ and $D_1=[-4.5,-0.5]$, $D_2=[-5.5,-1.5]$, $D_3=[1,4.5]$. $D_1$ and $D_2$ lie on the negative side while $D_3$ on the positive. By Theorem 4.2 we know that every $M \in [M]$ has exactly two negative eigenvalues $\in D_1\cup D_2$ and one positive $\in D_3$.

### RecIn test example

Consider the matrix,

$$\begin{aligned}{}[M] = \begin{bmatrix} [-0.08,0.88]&[0.30,0.55]&[0.65,0.84] \\ [0.30,0.55]&[-0.89,-0.69]&[-0.14,0.23] \\ [0.65,0.84]&[-0.14,0.23]&[-0.86,-0.74] \\ \end{bmatrix}. \end{aligned}$$

At the first step we have, $$[A^{(0)}] = [-0.89,-0.69] < 0 $$, $$[B^{(0)}]=[ \text { }[0.30,0.55] \text { }[-0.14,0.23]\text { }]$$ and $$[C^{(0)}]=\begin{bmatrix} [-0.08,0.38]&[0.65,0.34] \\ [0.65,0.34]&[-0.86,-0.74] \\ \end{bmatrix}$$.

For the second step, the (interval) Schur complement is

$$\begin{aligned}{}[M^{(1)}]=[C^{(0)}]-\frac{1}{[A^{(0)}]}{[B^{(0)}]}^T[B^{(0)}]=\begin{bmatrix} [0.02,0.81]&[0.23,0.83] \\ [0.23,0.83]&[-0.86,-0.66] \\ \end{bmatrix} \end{aligned}$$

and we have $[A^{(1)}]=[-0.86,-0.66]<0$ which is the second negative interval we find. Thus we conclude that every $M \in [M]$ has at least two negative eigenvalues.

### xRecIn test example

Consider this simple example,

$$\begin{aligned}{}[M] = \begin{bmatrix} [-2,-1]&0&0 \\ 0&[-1,1]&[2,3] \\ 0&[2,3]&[-1,1] \end{bmatrix}. \end{aligned}$$

If we were to apply RecIn, we would have at the first step: $[A^{(0)}] = [-2,-1] < 0$. However, at the next step, the (interval) Schur complement would be $$[M^{(1)}] = \begin{bmatrix} [-1,1]&[2,3] \\ [2,3]&[-1,1] \end{bmatrix}$$ and RecIn would not be able to proceed. Considering Corollary 4.6, we can set $$[M^{(1)}] = \begin{bmatrix} [1,1]&[2,3] \\ [2,3]&[1,1] \end{bmatrix}$$ and now we have $[A^{(1)}] = [1,1] > 0$ and the next Schur complement, $[M^{(2)}] = [1,1] - [4,9] / [1,1] = [-8,-3] < 0$. Thus every $M \in [M]$ has at least two negative eigenvalues.

### $2\times 2$ inertia test example

Consider the matrix from “RecIn test example”. At step 1 we have:

$$[M_{12}] = \begin{bmatrix} [-0.08,0.88]&[0.30,0.55] \\ [0.30,0.55]&[-0.89,-0.69] \\ \end{bmatrix}$$, $$\overline{\lambda _{12}}=1.05$$.

At step 2:

$$[M_{13}] = \begin{bmatrix} [-0.08,0.88]&[0.65,0.84] \\ [0.65,0.84]&[-0.86,-0.74] \\ \end{bmatrix}$$, $$\overline{\lambda _{13}}=1.24$$.

Finally, at step 3:

$$[M] = \begin{bmatrix} [-0.89,-0.69]&[-0.14,0.23] \\ [-0.14,0.23]&[-0.86,-0.74] \\ \end{bmatrix}$$ , $\overline{\lambda _{23}}=-0.48 <0$ and therefore we conclude that every $M \in [M]$ has at least two negative eigenvalues. With respect to the reverse of the Proposition 4.7, we can consider the following trivial counter-example:

$$\begin{aligned}{}[M] = \begin{bmatrix} [-2,-1]&0&100 \\ 0&[-2,-1]&0 \\ 100&0&0 \\ \end{bmatrix}. \end{aligned}$$

The sub-matrix $$[M_{12}] = \begin{bmatrix} [-2,-1]&0 \\ 0&[-2,-1] \\ \end{bmatrix}$$ clearly has two negative interval eigenvalues. The $2\times 2$ inertia test would identify this. However, because of the large entry, $m_{13} = 100$, the Gerschgorin discs would form one joint set with negative lower bound and positive upper bound and thus the Gerschgorin test would be inconclusive even for this very simple case.

### Rohn test example

Consider again the matrix from “RecIn test example”.

The center matrix is

$$\begin{aligned} C = \begin{bmatrix} 0.4&0.425&0.745 \\ 0.425&-0.79&0.045 \\ 0.745&0.045&-0.8 \\ \end{bmatrix}, \end{aligned}$$

and

$$\begin{aligned}{}[E] = \begin{bmatrix} [-0.44,0.44]&[-0.125,0.125]&[-0.095,0.095] \\ [-0.125,0.125]&[-0.10,0.10]&[-0.185,0.185] \\ [-0.095,0.095]&[-0.185,0.185]&[-0.06,0.06] \\ \end{bmatrix}. \end{aligned}$$

Since, $\overline{\lambda _2([M])} \le \lambda _2(C) + \overline{\lambda _1([E])}=-0.83 + 0.66 < 0$ we arrive at same conclusion as in “RecIn test example”.
